# Does density influence relative growth performance of farm, wild and F_1_ hybrid Atlantic salmon in semi-natural and hatchery common garden conditions?

**DOI:** 10.1098/rsos.160152

**Published:** 2016-07-06

**Authors:** Alison C. Harvey, Gareth Juleff, Gary R. Carvalho, Martin I. Taylor, Monica F. Solberg, Simon Creer, Lise Dyrhovden, Ivar-Helge Matre, Kevin A. Glover

**Affiliations:** 1 Molecular Ecology and Fisheries Genetics Laboratory, Bangor University, Bangor, Gwynedd LL57 2DG, UK; 2 Biological Sciences, University of East Anglia, Norwich, UK; 3 Havforskningsinstituttet, Bergen, Norway; 4 Sea Lice Research Centre, Universitetet i Bergen Institutt for Biologi, Bergen, Norway

**Keywords:** density, domestication, farm escapes, genetic interaction, growth, hybridization

## Abstract

The conditions encountered by Atlantic salmon, *Salmo salar* L., in aquaculture are markedly different from the natural environment. Typically, farmed salmon experience much higher densities than wild individuals, and may therefore have adapted to living in high densities. Previous studies have demonstrated that farmed salmon typically outgrow wild salmon by large ratios in the hatchery, but these differences are much less pronounced in the wild. Such divergence in growth may be explained partly by the offspring of wild salmon experiencing higher stress and thus lower growth when compared under high-density farming conditions. Here, growth of farmed, wild and F_1_ hybrid salmon was studied at contrasting densities within a hatchery and semi-natural environment. Farmed salmon significantly outgrew hybrid and wild salmon in all treatments. Importantly, however, the reaction norms were similar across treatments for all groups. Thus, this study was unable to find evidence that the offspring of farmed salmon have adapted more readily to higher fish densities than wild salmon as a result of domestication. It is suggested that the substantially higher growth rate of farmed salmon observed in the hatchery compared with wild individuals may not solely be caused by differences in their ability to grow in high-density hatchery scenarios.

## 1. Introduction

Captive populations undergo various morphological, physiological and behavioural changes during domestication [1]. Adaptation to the domestic environment occurs through two routes: environmentally induced changes to developmental processes within a single generation and genetic change across generations [2,3]. Relaxed natural selection can also result in domestic individuals that are more variable than wild conspecifics for certain traits that have adaptive value in the wild but less so in captivity [4]. For example, low mortality associated with domestic environments results in phenotypes persisting where they would not have persisted in the wild [5,6]. Genetic and morphological change occurs through direct and indirect responses to artificial selection and natural selection within the domestic environment contrasted with the wild environment (local adaptation in wild populations), and the differential mortality described above [2,5,6]. Random changes in allele frequencies can also arise through genetic drift in domestic populations with limited effective population sizes [4]. Thus, many domestic populations have become adapted to their captive environment, and may have reduced fitness in natural or novel environments when compared with wild individuals [3,4]. A loss of adaptive potential through domestication can negatively influence wild populations if domesticated individuals interbreed with wild conspecifics, such as when farmed individuals are released for restocking or are accidentally released through escape events.

Domesticated fish experience environments that differ vastly from those in nature in several ways [5,6]. Compared with the wild, hatchery environments typically display reduced environmental variation, fish densities are much higher, food is provided in excess, predation is absent, and there is no competition for mates [7,8]. Furthermore, there is often strong directional selection for a variety of commercially valuable traits such as growth rate and delayed maturation [9,10]. The outcome is that domestic fish are different from wild fish for several behavioural, morphological and physiological traits [5], likely underlain by genetically based as well as phenotypic plasticity [8].

Atlantic salmon (*Salmo salar,* Linnaeus (1758)) are iteroparous fish native to rivers on the east and west coasts of the Atlantic Ocean in the Northern Hemisphere [11]. They typically display an anadromous life cycle, although some populations spend their entire life cycle in freshwater. Stream-dwelling populations of wild Atlantic salmon typically exhibit territoriality [12], and individual growth and survival are regulated through exploitative (indirect competition for communal resources) and interference (direct resource competition through dominance or fighting) competition [13]. The density of salmon tends to vary greatly among and within river systems [14]. When densities are high, competition is exacerbated, and the population is regulated by density-dependent mortality, emigration or displacement [12]. Less commonly, the territory size of an individual will decrease, causing individual growth to decrease. Thus, population regulation occurs through density-dependent growth [13], though this type of population regulation is more common in lake-dwelling fish where emigration is not possible [12]. Studies show that when density in the wild is increased, individual growth decreases owing to density-dependent factors [12,15].

Growth is an important component of fitness [8], and body size is known to influence the outcome of social and resource competition [13,16]. Farmed Atlantic salmon have been under direct selection for fast growth for more than 10 generations, and consequently, the offspring of farmed salmon typically outgrow wild salmon by up to several fold under communal hatchery conditions [17–20]. In the wild, however, growth differences are far less pronounced [21–23]. The lower growth and survival of farmed fish within wild environments may be due to the high metabolic costs associated with increased aggression or maladapted foraging behaviour of farmed escapees [5], or their inability to adapt to variable feed in the natural environment [24]. Conversely, high growth differences observed between farmed and wild fish in the hatchery might derive from adaptation of farmed salmon to high densities, typically fed to excess. Reduced response to stress relative to their wild conspecifics has been documented in domestic salmon [19] and sea trout (anadromous *Salmo trutta* L.) [25]. While the increased stress, competition and social interaction associated with high densities would intuitively result in decreased growth as described above, it is thus possible that the domestication process has resulted in farmed strains that maintain high growth at high densities.

Understanding how changing environmental conditions such as density affect growth and survival in domestic and wild conspecifics, and their hybrids, can increase our knowledge of the risks associated with escapees of farmed fish and the consequences of hybridization. Here, a common garden design was used to investigate the growth of farmed, wild and F_1_ hybrid Atlantic salmon offspring at three contrasting densities within a hatchery, and at two contrasting densities under semi-natural conditions. The aim was to investigate whether differences in growth rates between farmed, wild and F_1_ hybrid salmon displayed similar reaction norms at different densities in the two environments. Specifically, the hypothesis tested was that the relative growth difference between farmed and wild salmon would be higher in the high-density conditions as a result of adaptation of farmed salmon to those conditions.

## 2. Material and methods

### 2.1. Family production

All families used in this experiment were established in November 2013 at Matre, the Institute of Marine Research's experimental fish farm in Norway. Atlantic salmon from the commercial farmed strain Mowi and wild-caught Atlantic salmon from the River Etne (59°40′N, 5°56′E) were used to produce five pure farmed, five pure wild and five F_1_ hybrid families (15 families in total; electronic supplementary material, table S1). Mowi is the oldest Norwegian commercial strain and is used by Marine Harvest [26]. Mowi was established in the late 1960s primarily using fish from the River Vosso and the River Aaroy, whose populations are known to contain large multi-sea winter fish [18]. The main traits that have been under selection in the Mowi strain are growth, late maturation and fillet quality. The farmed salmon used in this study had undergone over 10 generations of selection. The salmon population from the River Etne is the largest salmon population in Hordaland, western Norway. A 2004 report estimated that the smolt production for the River Etne was around 30 000 individuals in a 15 km^2^ area [27]. A study conducted using snorkelling observations and catch statistics for the period 2004–2011 estimated that the median number of wild fish in the Hardangerfjord river system (including the River Etne) was estimated to be 3.5 fish per 10 000 m^2^ [28]. The wild parental salmon were collected directly from the river in the autumn of 2013 by angling and transferred to the local hatchery where they were held until gametes were stripped from the fish. Fish scales were read from these individuals in order to ensure that they were wild fish and not farmed escapes [29]. Population genetic analyses have revealed introgression of farmed salmon in a number of Norwegian populations, including the population in the River Etne [30,31]. Therefore, although the wild fish used in this study were indeed born in the wild (based upon scale reading), it is not possible to completely exclude the possibility that some of those individuals used as broodstock may have admixed ancestries at some level.

All F_1_ hybrids were produced by crossing a farmed Mowi female with a wild Etne male (Mowi × Etne). The hybrids were thus maternal and paternal half-siblings with the farmed and wild families, respectively. From here on, group refers to the origin of each cross-type, i.e. farmed, wild and hybrid.

Eyed eggs were sorted into hatchery trays representing the treatment replicates in week 5 of 2014 (where week 1 = first week of January). The replicates were all incubated under standard hatchery conditions until transfer to tanks. Dead eggs were removed when necessary. In the hatchery treatments, the control and high-density replicates initially consisted of 30 eggs each from the 15 families (*n* = 450 per tank), whereas each low-density replicate consisted initially of 15 eggs from each family (*n* = 225 per tank). In the semi-natural treatments, the low-density replicates consisted of 30 eggs from each family (*n* = 450 per tank), and the high-density replicates consisted of 90 eggs per family (*n* = 1350 per tank). Egg volume measurements were taken from each family in order to calculate average family egg diameter. Egg diameter was calculated as 25 cm divided by the number of eggs counted on a 25 cm rule.

### 2.2. Experimental design

In order to investigate the effect of density and environment on growth and survival in salmon of farmed, wild and hybrid origin, fish were reared in communal fish tanks (i.e. common garden) at three densities in a hatchery environment and at two densities in a semi-natural environment. These treatment densities were chosen to represent densities that farmed and wild fish may not typically experience in their respective local environments, where typically the farming environment is characterized by much higher densities than the wild environment. For an overview of the experiment, see table 1. The treatments consisted of five differing rearing conditions: three hatchery treatments further differentiated into high, low and control densities, and two semi-natural treatments consisting of high and low densities. Treatment from here on refers to the five different rearing conditions as described below.

**Table 1.** Details of the experimental design. Initial numbers of eggs per family within each replicate treatment and the water level and volumes of each treatment.

	hatchery			semi-natural	
treatment	low	control	high	low	high
replicates (*n*)	2	2	2	2	2
initial fish per replicate	225	450	450	450	1350
families per replicate	five farmed: five hybrid: five wild in all treatment replicate tanks				
total fish	450	900	900	900	2700
water level (cm)	55	55	13.5	25	25
volume (m^3^)	1.2375	1.2375	0.30375	7.85	7.85

#### 2.2.1. Hatchery treatments

Three treatments were set up within a hatchery environment to represent (i) low density (approx. 0.16 fish l^−1^), (ii) a control density (approx. 0.36 fish l^−1^) which represented a standard hatchery density, and (iii) a high-density environment (approx. 1.5 fish l^−1^). These are hereon referred to as the low, control and high hatchery treatments. Each treatment consisted of two replicate tanks with six experimental hatchery tanks in total. The low-density treatment was established by initially using half the number of fish used in the control and high treatments. The high-density treatment consisted of the same initial number of fish as the control treatment with a water level 25% of the control water level (55 cm) to simulate a high-density environment.

Unfed fry were transferred from the hatchery incubators to the experimental tanks in week 17, when treatment conditions commenced. The fish were reared in 1.5 m^2^ tanks with a maximum flow rate of 35 l min^−1^ at ambient water temperature. Temperature was recorded daily and ranged from 4.5 to 14.4°C. Start feeding began in week 18, and fish were fed a commercial pellet diet (Skretting) ad libitum. Pellet size was adjusted according to manufacturer's tables, and the fish were kept on 24 h photoperiod throughout the experiment as is standard in salmon hatcheries.

#### 2.2.2. Semi-natural treatments

The semi-natural environment consisted of replicate doughnut-shaped 7.85 m^3^ tanks (outer diameter 7 m, inner diameter 3 m) filled with gravel (of variable sizes to reflect a natural river bed, no larger than approx. 30 cm in diameter) and situated outdoors [20,32]. Water level was kept at 25 cm in both treatments. The density conditions were imposed by adding three times as many fish into the high-density treatment (approx. 0.11 fish l^−1^) compared with the low-density treatment (approx. 0.05 fish l^−1^). The treatments are from here on referred to as the low and high semi-natural treatments. Each treatment consisted of two replicate tanks; therefore, there were four experimental semi-natural tanks.

Fish were planted out as fry into the semi-natural environment in week 17, when treatment conditions commenced. Automatic feeders were situated near the water inlet and fish were fed ad libitum as in the hatchery experiment. The fish experienced natural light conditions and ambient water temperature which ranged from 4.6 to 14.4°C across the experimental period (electronic supplementary material, figure S1). Average daily temperature was used to calculate the degree days for the hatchery and semi-natural treatments. The semi-natural tanks were predator-free, i.e. no predators were explicitly placed within the tanks.

### 2.3. Sampling and data

**Figure 1.**
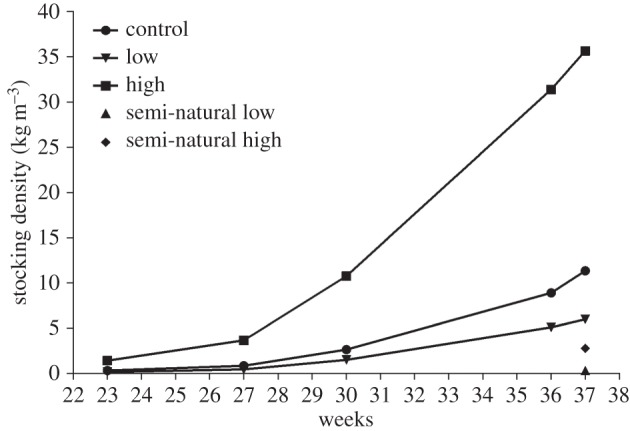
Average stocking density of the treatments. The stocking density was calculated by estimating average biomass per replicate by weighing a random sample of 100 fish from each tank at specific time points within the experiment duration. This was only possible for the hatchery tanks, and therefore, only the stocking density at experiment termination is presented for the semi-natural tanks.

The experiment ran for 20 weeks and was terminated in calendar week 37 of 2014. Mortality was recorded daily for each hatchery treatment replicate and was used to estimate total mortality at experiment termination. Average biomass within the hatchery treatments was estimated each month by measuring 100 randomly sampled fish in each replicate, which allowed for the estimation of stocking density within these treatments as the experiment progressed (figure 1). It was not possible to record daily mortality in the outdoor semi-natural tanks; however, total mortality was estimated using the number of surviving fish sampled at the end of the experiment. Mortality data are presented in table 2. All remaining fish were euthanized with an overdose of Finquel Vet anaesthetic following standard guidelines (Årnes, Norway). Individual growth measurements of wet weight and fork length were recorded, and adipose or caudal fin samples were taken from each individual and stored in 100% ethanol. A total of 2105 individuals were sampled in the hatchery tanks, and a total of 1883 individuals were sampled in the semi-natural tanks.

**Table 2.** Weight, mortality and average densities within treatments at experiment termination. High and low correspond to the density of fish in the treatments, whereas control represents an intermediate density. First and last correspond to the first density calculated from average biomass per treatment taken in week 23 and the final density measurement calculated from final weight data taken in week 37.

			weight (g)		density (kg 1000 l^−1^)		
treatment	tank	*n*, sampled	mean	s.d.	first	last	mortality (%)
hatchery low	1	205	35.06	11.28	0.16	5.99	9.9
	2	212	36.03	11.1			5.8
hatchery control	3	421	33.01	11.16	0.32	11.35	6.5
	4	422	33.72	11.94			6.7
hatchery high	5	424	26.07	9.78	1.41	35.66	5.8
	6	421	25.2	8.57			6.5
semi-natural low	7	85	16.92	7.89	n.a.	0.33	81.2
	8	98	11.29	5.63			78.3
semi-natural high	9	861	13.79	6.02	n.a.	2.8	36.3
	10	839	12.01	5.37			37.9

### 2.4. Genotyping and parentage assignment

DNA-based parentage testing was used to assign individual fish from the hatchery and semi-natural treatments respectively back to their family of origin. DNA was extracted in 96-well plates using a variation of the salt extraction method [33]. Parental DNA was extracted and genotyped twice to ensure consistent genotyping. Each plate contained two randomly placed negative controls (blank wells) to ensure unique identification of each plate. Five microsatellites were amplified in a single PCR multiplex: *SsaF43* [34], *Ssa197* [35], *SSsp3016* (GenBank no. AY372820), *MHCI* [36] and *MHCII* [37]. There were 38 individuals from the hatchery experiment and 82 individuals from the semi-natural experiment that could not be unambiguously assigned back to one family using the original multiplex. These samples were genotyped using additional loci (electronic supplementary material, table S2) in order to unequivocally identify their families. PCR products were resolved on an ABI Applied Biosystems 3731 Genetic Analyser and sized using a 500LIZ standard (Applied Biosystems). Genemapper v. 4.0 was used to score alleles manually. Individuals were then assigned back to family using the Family Analysis Program (v. 3.6) [38].

### 2.5. Statistical analysis

Statistical analysis was carried out using R v. 3.1.3 [39], and all critical *p*-values were set to 0.05, unless otherwise stated.

#### 2.5.1. Growth

A linear mixed-effect (LME) model was used to investigate the variation in weight at termination. The response variable was the continuous variable of log-transformed weight at termination. The LME model was fitted using *lmer* from the lmerTest package in R [40]. The full model was fitted with treatment (T) and group (G) as fixed categorical factors, egg size (E) as a continuous fixed effect, and all two-way interactions between the fixed covariates: treatment and group (TG), treatment and egg (TE) and group and egg (GE) as fixed effects. Tank replicates (t) nested within treatments were included as a random intercept effect (10 levels), and family (f) was included as a random intercept effect (15 levels) with differing slopes for the effect of treatment

2.1 $$Y=\beta_{0}+\beta_{1}T+\beta_{2}G+\beta_{3}E+\beta_{4}TG+\beta_{5}TE+\beta_{6}GE+b_{t}+b_{f}+\varepsilon ,\;\text{where}\;\varepsilon \tilde{N}(0,\;\sigma^{2}),$$

where *β*_0_ is the intercept and *ϵ* is the normally distributed error term. The lmerTest package in R allows for automatic model selection using the *step* function [40]. The function performs backwards selection on both the fixed and random effects to determine the simplest best-fitting model [40]

2.2 $$Y=\beta_{0}+\beta_{1}T+\beta_{2}G+\beta_{3}E+\beta_{4}TE+b_{t}+b_{f}+\varepsilon ,\;\mathrm{where}\;\varepsilon \tilde{N}(0,\;\sigma^{2}).$$

It first performs backwards selection on the random elements of the model using likelihood ratio tests, with a significance level of 0.1 as a default, before performing backwards selection on the fixed elements in the model [40]. The *p*-values generated for the fixed part of the model are calculated, using an *F*-test based on the Satterthwaite approximation, and the significance level is set to 0.05 [40]. Both the full and final model fits were confirmed by investigating the plots of the model residuals against the covariates included in the model as well as those that were not included in the model. Normality of the model residuals was confirmed visually using histograms. The full and final model with parameter estimates as given by the *lme4* output with overall covariate *p*-values generated from the *step* function is presented in table 3. Pairwise comparisons of log weight between treatments and between groups were performed using the *glht* function in the *multcomp* package with Tukey adjustments for multiple comparisons (electronic supplementary material, table S3) [41]. Pairwise comparisons of egg size among the groups were performed, using the *glht* function as above (electronic supplementary material, table S3). Relative growth differences comparing the average weight in grams and log weight of farmed to wild and hybrid to wild fish are presented for each treatment in table 4.

**Table 3.** Parameter estimates of the full model for the linear mixed model investigating log weight variation. The final model (equation (2.2)) covariates are presented in italics. The final column gives single *p*-values estimated for each covariate in the full model using the step function in the lmerTest package by an *F*-test based on the Satterthwaite approximation. The significance level is set to 0.05 unless otherwise stated. S.e., standard error of the parameter estimates; s.d., standard deviation of the variance estimates of the random effects.

covariate	fixed effects	parameter estimate	s.e.	*t*-value	*p*-value	overall *p*-value
	intercept	1.64	0.05	35.51	0.00	
*treatment*	*hatchery control*	−*0*.*02*	*0*.*06*	−*0*.*35*	*0*.*74*	0.00
	*hatchery high*	−*0*.*12*	*0*.*06*	−*2*.*01*	*0*.*09*	
	*semi-natural high*	−*0*.*45*	*0*.*06*	−*7*.*12*	*0*.*00*	
	*semi-natural low*	−*0*.*401*	*0*.*07*	−*5*.*92*	*0*.*00*	
*group*	*hybrid*	−*0*.*07*	*0*.*04*	−*1*.*99*	*0*.*07*	*0*.*00*
	*wild*	−*0*.*27*	*0*.*04*	−*7*.*60*	*0*.*00*	
*egg size*	*egg size*	−*0*.*02*	*0*.*03*	−*0*.*68*	*0*.*51*	*0*.*00*
treatment × group	hatchery control × hybrid	−0.01	0.03	−0.25	0.80	0.58
	hatchery high × hybrid	−0.01	0.03	−0.16	0.87	
	semi-natural high × hybrid	0.00	0.04	−0.01	0.99	
	semi-natural low × hybrid	0.03	0.06	0.45	0.66	
	hatchery control × wild	−0.03	0.03	−1.03	0.32	
	hatchery high × wild	−0.09	0.04	−2.31	0.04	
	semi-natural high × wild	−0.05	0.05	−1.11	0.29	
	semi-natural low × wild	−0.05	0.06	−0.90	0.39	
*treatment × egg size*	*hatchery control × egg size*	*0*.*01*	*0*.*01*	*1*.*07*	*0*.*30*	*0*.*03*
	*hatchery high × egg size*	*0*.*03*	*0*.*02*	*2*.*22*	*0*.*05*	
	*semi-natural high × egg size*	*0*.*07*	*0*.*02*	*3*.*40*	*0*.*01*	
	*semi-natural low × egg size*	*0*.*07*	*0*.*02*	*2*.*91*	*0*.*01*	
group × egg size	hybrid × egg size	0.05	0.04	1.27	0.24	0.48
	wild × egg size	0.30	0.03	0.90	0.39	

	random effects	variance	s.d.			
family	hatchery low	0.002	0.044			
	hatchery control	0.001	0.025			
	hatchery high	0.002	0.041			
	semi-natural high	0.004	0.065			
	semi-natural low	0.004	0.062			
tank		0.003	0.055			
residual		0.020	0.150

**Table 4.** Relative weight and log weight differences between each group within each treatment. The relative growth differences were calculated by dividing the average weight (*W*) in grams of the farmed fish by the wild and hybrid fish respectively, and the average weight of the hybrid fish by the wild fish within each treatment. The relative log weight (log *W*) differences were calculated as above using the log weights of each group within each treatment.

			relative *W* (g) difference			relative log *W* difference	
treatment	origin	*W* (g)	to wild	to hybrid	log *W*	to wild	to hybrid
hatchery							
low	farm	45.2	1.8	1.2	1.64	1.2	1.1
	hybrid	36.45	1.5		1.55	1.1	
	wild	24.74			1.37		
control	farm	42.95	1.9	1.2	1.62	1.2	1.1
	hybrid	34.53	1.5		1.52	1.2	
	wild	22.71			1.32		
high	farm	33.51	2	1.2	1.51	1.3	1.1
	hybrid	26.85	1.6		1.41	1.2	
	wild	16.68			1.19		
semi-natural							
low	farm	19	2	1.3	1.24	1.3	1.1
	hybrid	15.04	1.6		1.13	1.2	
	wild	9.3			0.92		
high	farm	16.68	1.9	1.3	1.19	1.3	1.1
	hybrid	13.15	1.5		1.08	1.2	
	wild	8.99			0.92		

#### 2.5.2. Mortality

In order to investigate whether survival differed between treatments, a generalized linear-mixed effect model (GLMM) was fitted using the *glmer* function in the lme4 package [42]. The full model included the fixed factor covariates of treatment (T) and group (G), the continuous effect of egg size (E) and two-way interactions between the fixed covariates: treatment and egg (TE), treatment and group (TG) and group and egg size (GE). In order to control for any differences in mortality between replicates and families the variables tank (t) and family (f) were included in the model as random intercept covariates

2.3 $$\mathrm{logit}(Y)=\beta_{0}+\beta_{1}T+\beta_{2}G+\beta_{3}E+\beta_{4}TE+\beta_{5}TG+\beta_{6}GE+b_{t}+b_{f}\;+\varepsilon ,\;$$

where *β*_0_ is the intercept and *ϵ* is the error term. The response variable, survival, was binary, and thus the binomial distribution was used with the default logit link function, and the model was fitted using the Laplace approximation. The random effect structure was investigated by fitting the full model with only one random effect at a time and plotting the 95% prediction intervals of the random effect using the *dotplot* function in the lattice package [43]. If all the prediction intervals of the random effect overlapped zero, then this effect was removed from the final model. The mixed function from the afex package was used to investigate the significance of the fixed covariates [44]. The function calculates type 3-like *p*-values for each fixed covariate based on parametric bootstrapping [44]. Parameter estimates and the *p*-values of the fixed effects are presented in table 5. The final model included covariates that yielded the best fit

2.4 $$\mathrm{logit}(Y)=\beta_{0}+\beta_{1}T+\beta_{4}TE+b_{f}+\varepsilon .$$

**Table 5.** Parameter estimates of the GLMM investigating variation in survival and overall *p*-values of each model covariate. The final column gives single *p*-values estimated for each covariate within the final model estimated using the *mixed* function in the afex package by parametric bootstrapping. Covariates in italics were retained in the final model. S.e., standard error of the parameter estimates of the fixed effects; s.d., standard deviation of the variance estimates of the random effects.

covariate	fixed effects	parameter estimate	s.e.	*Z*-value	*P*-value	overall *p*-value
	intercept	2.46	0.33	7.56	0.00	
*treatment*	*hatchery high*	*0*.*18*	*0*.*31*	*0*.*59*	*0*.*55*	*0*.*00*
	*hatchery low*	−*0*.*73*	*0*.*36*	−*0*.*20*	*0*.*84*	
	*semi-natural high*	−*1*.*97*	*0*.*22*	−*8*.*79*	<*2* × *10*^−*16*^	
	*semi-natural low*	−*4*.*40*	*0*.*27*	−*16*.*13*	<*2* × *10*^−*16*^	
group	hybrid	1.08	0.52	2.08	0.04	0.59
	wild	−0.03	0.46	−0.07	0.95	
egg size	egg size	−4.90	80.79	−0.06	0.95	0.86
treatment × group	hatchery high × hybrid	−0.19	0.51	−0.38	0.71	0.08
	hatchery low × hybrid	0.21	0.66	0.32	0.75	
	semi-natural high × hybrid	−0.78	0.38	−2.06	0.04	
	semi-natural low × hybrid	−0.43	0.43	−1.01	0.31	
	hatchery high × wild	0.13	0.45	0.28	0.78	
	hatchery low × wild	−0.27	0.51	−0.53	0.60	
	semi-natural high × wild	−0.07	0.33	−0.22	0.83	
	semi-natural low × wild	0.75	0.38	1.97	0.05	
*treatment* × *egg size*	*hatchery high* × *egg size*	*53*.*54*	*31*.*76*	*1*.*69*	*0*.*09*	*0*.*00*
	*hatchery low* ×*egg size*	−*20*.*76*	*32*.*41*	−*0*.*64*	*0*.*52*	
	*semi-natural high* × *egg size*	−*4*.*02*	*21*.*54*	−*0*.*19*	*0*.*85*	
	*semi-natural low* × *egg size*	−*84*.*36*	*25*.*48*	−*3*.*30*	*0*.*00*	
group × egg size	hybrid × egg size	74.19	107.60	0.69	0.49	0.89
	wild × egg size	47.56	82.28	0.58	0.56	

	random effects	variance	s.d.			
	tank	0.00	0.00			
	*family*	*0*.*24*	*0*.*49*			
	*deviance*	5331.30				

## 3. Results

### 3.1. Genotyping and parentage assignment

Of the 3988 individuals sampled, 11 individuals (less than 0.001% of the total) could not be assigned unambiguously back to a single family using the microsatellite multiplexes. A further four individuals were identified as outliers owing to extreme condition factors attributed to human recording error and subsequently removed from the dataset prior to analysis. Thus, a total of 3973 individuals were used in the analysis.

### 3.2. Statistical analysis

#### 3.2.1. Growth

**Figure 2.**
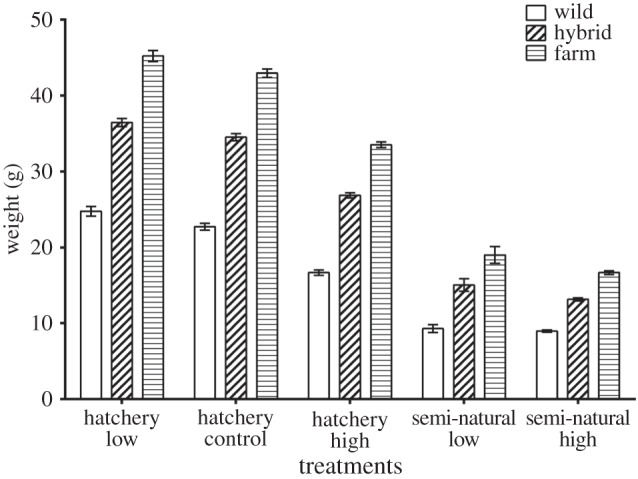
Average weights of each group within each treatment. Bars represent the standard error of the mean weight of each group within the treatments.

**Figure 3.**
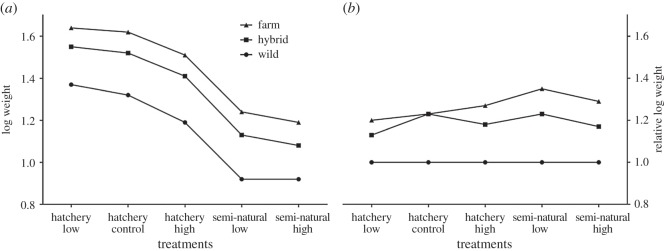
(*a*) Phenotypic growth reaction norms for each group across the treatments (average log weight) and (*b*) the average log weights relative to the wild group. In panel (*b*), the hybrid and farmed groups are compared with the wild group within each treatment. The *x*-axis shows the treatments.

Treatment, group, egg size and the interaction of egg size and treatment were retained as significant effects in the growth model (table 3). All genetic groups grew significantly different from each other across the treatments, with farmed fish being larger than hybrid and wild fish, and hybrid fish being larger than wild fish (electronic supplementary material, table S3; figure 2). On average, all fish grew larger in the hatchery density treatments, and growth of all groups was lowest in the semi-natural density treatments (figure 2). The interaction between treatment and group was not significant, indicating that all groups responded equally relative to the other groups across the treatments, indicated by the similar relative growth differences in table 4 and the reaction norms in figure 3. Within the hatchery treatments, growth of all three genetic groups decreased as density increased, with the lowest growth observed in the high-density hatchery treatment, although the difference in growth between the hatchery treatments was not significant (electronic supplementary material, table S3). Similarly, growth was not significantly different between the two semi-natural treatments, although it was visibly lowest in the semi-natural high-density treatment (figure 2). The final model (equation (2.2) and table 3) retained an effect of egg size and a significant interaction between egg size and treatment. Egg size was significantly different among the groups (electronic supplementary material, table S3) and was found to be negatively correlated to weight. It was found that egg size was only a significant predictor of weight in the semi-natural treatments, as the fish in these treatments displayed the lowest weights, possibly owing to a slower development compared with the hatchery treatments (electronic supplementary material, table S4). There was a difference in degree days between the hatchery (1796 degree days) and the semi-natural treatments (1586 degree days) owing to different ambient temperatures between the indoor (hatchery) and outdoor (semi-natural) tanks (electronic supplementary material, figure S1). Egg size was also significant in the hatchery high-density treatment, where growth was also low. The random effects of tank replicate and family were retained in the final model in order to control for any variation within these variables.

#### 3.2.2. Mortality

**Figure 4.**
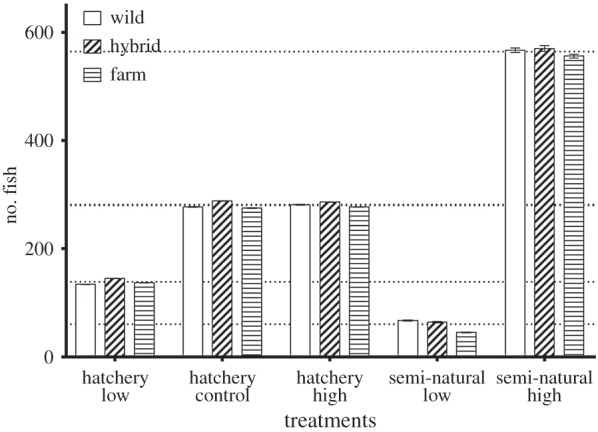
Average number of fish surviving for each group within each treatment. Dotted horizontal lines represent the expected number of surviving fish per group in each treatment based on average mortality. Error bars represent the standard error of the average family variation per group within each treatment.

Percentage survival was highest in the hatchery treatments, with no significant differences among treatments observed (table 2 and figure 4). Within the semi-natural treatments for all groups, survival was highest in the high-density treatment (table 2 and figure 4). The low survival observed in the semi-natural low-density treatment was not a result of high mortality in one specific replicate: the random effect of tank was excluded from the final model owing to its non-significant effect; therefore, mortality was insignificantly different between replicates within each treatment. The final model retained a significant effect of treatment and an interaction between egg size and treatment, whereas egg size alone was not significant (equation (2.4) and table 5). On further analysis of the data split into each treatment, it was found that egg size was only significant in the hatchery high-density treatment (electronic supplementary material, table 5).

## 4. Discussion

Growth and survival of fish are influenced by density and availability of food [45,46]. The offspring of farmed Atlantic salmon generally outgrow wild salmon twofold or more under hatchery conditions [18–20], possibly owing to adaptation to high densities through domestication. Therefore, it was hypothesized that farmed salmon may be able to maintain higher growth than their wild conspecifics in high-density environments, potentially explaining the elevated growth differences observed between farmed and wild conspecifics under hatchery conditions. Here, it was found that density influenced growth of all genetic groups equally, with all groups exhibiting decreased growth at higher densities; farmed salmon had the highest average growth within each treatment while wild fish had the lowest growth within each treatment; and the mortality of all groups was similar for all the treatments. Thus, this study was unable to find evidence of adaptation of farmed fish to high densities using the present treatment densities, tentatively suggesting that high-density adaptation is not driving the divergence in growth observed between farmed and wild salmon under hatchery conditions.

### 4.1. Growth

High-density conditions are known to lead to behavioural changes, induce stress behaviours and lower feed utilization, all of which can decrease growth among fish [47]. Refstie & Kittelsen [45] found that under controlled conditions with excess feed the growth of two domesticated populations of Atlantic salmon decreased as density increased. The negative effect of higher densities on growth has also been observed in other fish species [15,48], and has been attributed to an increase in intraspecific competition and agonistic behaviour at high densities [12,49]. In natural systems, density-dependent growth will also be controlled by the number of predators and by the competition for limited resources [13]. As there were no predators in this study, and the available food was not limiting, it is likely that the lower growth observed at the higher densities could be the result of higher stress in all groups which may have caused the fish to feed less effectively relative to the other treatments.

Growth and body size is an important factor determining competition and reproductive success [50]. Directional selection for growth has resulted in farmed salmon displaying higher growth rates than wild salmon when compared under hatchery conditions [19], and this growth may give the offspring of escaped farmed salmon a competitive advantage over wild conspecifics in the wild, although often the growth differences observed in the wild are much lower [21,22]. Under standard hatchery conditions, the relative growth differences between farmed and wild conspecifics have been documented to be as high as threefold [19] and even fivefold [20], with Glover *et al.* [18] observing that farmed salmon were twice as large as their wild conspecifics at the end of a full aquaculture production cycle. In this study, farmed salmon grew significantly larger than wild salmon in all treatments, although this growth difference was much lower than previously observed in a hatchery study using the same strains [19]. Interestingly, Reed *et al.* [23] reported relatively moderate differences (5–20%) between farmed and wild salmon parr for size-at-age in the wild, and they found that their observed growth differences were similar in the hatchery environment as in the wild, contrasting the results of previously cited studies. They attribute these differences to the difference in historical selection regimes and generation time between the farmed strain used in their study (Irish farm strain derived from the Norwegian Mowi strain in 1983) compared with the other studies (more recent Norwegian Mowi strain) [23].

Solberg *et al.* [19] found that juvenile farmed salmon exhibited a lower response to stress than their hybrid and wild conspecifics when exposed to a twice-daily stressor of lowered water levels, indicating that domestication has resulted in farmed salmon which are able to maintain a higher level of growth under stressful conditions. Elevated stress due to crowded conditions has been shown to negatively influence appetite and growth performance in Atlantic salmon [51] and brown trout [52]. It is possible that the process of domestication may have adapted farmed salmon to higher growth under stressful high-density conditions. Thus, farmed salmon in the present study would be expected to maintain a higher growth relative to the wild salmon at high densities within the hatchery treatment. However, this was not the case here. No evidence was found for an interaction between group and treatment (genotype × environment interaction; table 3), and the similar relative growth differences between groups among the treatments indicate that each group is responding to the treatments similarly relative to the other groups (table 4 and figure 3). It is acknowledged that the treatments used in this study may not have been different enough to elicit a growth divergence response owing to density adaptation; however, the findings suggest that the higher growth differences observed in the hatchery are probably not the result of farmed fish being more adapted to growth at higher densities than wild fish.

The ability of an individual to adapt its behavioural strategy (plasticity) can influence fitness and competition [53]. Many salmonids exhibit behavioural plasticity depending on the circumstance, for example exhibiting territorial behaviour in low densities, and schooling behaviour in high densities [53,54]. At certain densities, it becomes too metabolically costly to defend a territory [53,54]. Under controlled conditions, Brännäs *et al.* [53] found that interspecific competition among stocked brown trout depended on a variety of factors, including competitive ability, food availability and prior residency. They found that growth of all groups was depressed at higher densities and it was advantageous to be less aggressive at high densities and also to be a larger individual [53]. Farmed salmon are generally observed to be more aggressive than wild salmon, possibly inadvertently through selection for increased growth or because they have not been able to establish social or dominance hierarchies under hatchery conditions and may not understand the trade-off between aggression and its energetic cost in certain situations [5]. Higher levels of growth hormone may also influence aggression in salmonids [55], and may also affect foraging behaviour and metabolic demands [16]. These behavioural and hormonal changes within farmed salmon may partly explain their lower relative growth observed in the wild. Solberg *et al.* [20] found that growth differences between farmed and wild conspecifics decreased along an environmental gradient from hatchery to semi-natural conditions with restricted feed. They suggest that the lower growth observed in wild studies could be caused by a combination of negative and positive size-selective mortality, whereby faster-growing individuals can outcompete smaller individuals for resources (negative size selection) and where faster-growing individuals are more prone to predation over smaller individuals (positive size selection), resulting in fish of all origins being of a similar size (although positive size selection was not explicitly tested in their study) [20].

In this study, growth was low among all groups in the semi-natural treatments (figure 2), despite these two treatments having the lowest densities among all treatments. In the wild, salmonids are territorial and establish a social hierarchy among individuals that influence individual growth, with low-ranking fish having reduced access to feed and displaying reduced growth relative to the dominant individuals [49]. If the semi-natural environment induced territorial or dominance effects among the fish, one would then expect to see distinct size classes representing the larger, dominant fish and the smaller, less dominant individuals. However, such trends were not observed. There was a difference in degree days between the hatchery and semi-natural treatments; therefore, it is likely that other environmental conditions such as the naturally varying water temperature or ambient light conditions were responsible at least partly for the low growth observed in the semi-natural treatments. It is possible that the densities imposed on these semi-natural tanks were not sufficient to affect growth among the groups. Jørgensen *et al.* [49] investigated the effects of density on hatchery-reared Arctic charr (*Salvelinus alpinus* L.) under controlled conditions. Interestingly, they found depressed growth rates in the low-density treatment, and observed schooling behaviour of fish in their medium and high-density tanks [49]. In this study, schooling behaviour was observed within the high-density semi-natural replicates. While the low water temperature is probably the main reason behind the low growth observed in the semi-natural treatments, it is possible that the increased swimming behaviour and social interaction may have influenced growth.

In similar comparative studies of Atlantic salmon, hybrids often display intermediate levels of growth compared with their farmed and wild parental strains [18,20,55,56]. Hybrid vigour commonly occurs when one or both of the parental strains are inbred, whereas a decreased performance observed in hybrids relative to their parents may occur via outbreeding depression [55]. In this study, hybrids grew significantly different from both wild and farmed conspecifics; however, there was an observable non-significant trend of hybrid relative growth being more similar to their farmed parents in each treatment (1.5–1.6 : 1 for hybrid to wild and 1.2–1.3 : 1 for farmed to hybrids using the average raw weight in grams; table 4 and figure 3). A study which used the same parental strains as this study also observed intermediate hybrid growth and the same trend of more similar growth with the farmed parents [19]. It is not thought that the growth levels observed in this study represent hybrid vigour, as the relative growth differences are still somewhat intermediate (table 5 and figure 3) and growth was significantly different among the groups (electronic supplementary material, table S3), indicating additive effects. It is acknowledged, however, that a more complete hybrid group design (i.e. reciprocal crosses) would allow for the unambiguous conclusion of additive hybrid growth effects. Several studies comparing gene transcription between farmed and wild salmonids observed some level of non-intermediate (non-additive) gene expression in hybrids [57–59], and this may be population specific [60,61]. Bicskei *et al.* [58] examined gene transcription in farmed, F_1_ hybrid and wild Atlantic salmon at two life stages, and found fewer significantly differentially expressed transcripts between farmed and hybrid individuals than between hybrid and wild individuals. Their hybrid crosses were generated from the farmed females, and suggest that maternal effects might account for this bias [58]. They found that the heritability patterns of many of the differentially expressed transcripts in the hybrid fish were either intermediate or maternally dominant [58], highlighting the need for reciprocal hybrid crosses in comparative studies. Maternal effects, such as egg size or maternal body size, can greatly influence offspring development and fitness [62]. Often maternal effects are taken into account in order to avoid overestimating or confusing genetic effects with environmental maternal effects [63]. In the present study, the maternal effect of egg size was controlled for by including it as a covariate in the growth model.

Overall, egg size was found to be negatively influencing growth, owing to the larger average egg sizes of the wild families used in the present study coupled with their lower growth compared to the farmed and hybrid families. Generally, a larger egg size is expected to convey a positive size advantage to offspring [64]; however, negative maternal effects have been observed in Chinook salmon (*Oncorhynchus tshawytscha* L.), whereby the initial positive effect of large egg size on growth was reversed after a period of time [63]. The authors attribute this switch in egg size effect to variation in growth rate among families with different egg sizes [63]. In the present study the growth model identified the interaction between egg size and treatment as a significant predictor of growth (equation (2.2) and table 4), and when egg size was included in the models for growth at each treatment, it was found that it was only significant in the semi-natural treatments and the hatchery high-density treatment. It is possible that the lack of degree days meant the smaller fish had had less time to develop and had not yet overcome the effect of egg size, which is known to decrease with offspring development [65].

### 4.2. Mortality

Mortality within the hatchery treatments was low, and did not differ between treatments or between the groups (figure 4). There was high mortality observed within the low-density semi-natural replicates (81.2% and 78.3%; table 2), and moderate mortality within the high-density semi-natural tanks (36.3% and 37.9%; table 2). It is not possible to determine when the majority of this mortality occurred, or whether it was a gradual or acute event. It is therefore not possible to say how this may have influenced growth as the experiment continued. In natural conditions, salmonids are territorial [13] and this may impose a density-dependent effect on mortality. Within a stream environment as population density increases past the carrying capacity for territories several processes can occur: territory size may decrease and influence growth through density dependence or those who are unable to acquire a territory and access to food may emigrate or die [12,13]. Generally, mortality is observed to be positively related to stocking density [49]; therefore, it is unclear why it was the low-density semi-natural replicates that suffered such high mortalities. There was no effect of group origin on mortality, indicating that all groups suffered similar relative mortalities (figure 4). Interestingly, both replicates from each of the semi-natural treatments experienced similar mortality, indicating no influence of tank effects on mortality (equation (2.4) and figure 4). There was no observed predation from birds (I.-H.M. 2014, personal communication). The mortality model identified treatment and the interaction between treatment and egg size as predictors of survival (equation (2.4) and table 5). When the effect of egg size on mortality was investigated for each treatment, it was found that egg size was only significant within the high-density hatchery treatment.

### 4.3. General implications

While comparing the relative growth of farmed, hybrid and wild salmon families under different densities, there was no evidence found to suggest that farmed salmon have adapted to higher stocking densities. Although the possibility cannot be excluded that higher and lower densities than those used in this study may elicit such effects, our treatments, nevertheless, elicited a response in modifying growth of all salmon reared here. The lack of interaction between density and relative growth of farmed, hybrid and wild salmon observed here suggests that differences in relative growth between farmed, hybrid and wild salmon between the hatchery environment and the wild is caused by a complex of other factors, and not attributable mainly to density. Competitive experiments in the wild at differing densities have suggested that farmed salmon display relatively greater mortality than wild salmon under higher densities [22], and population genetic studies have demonstrated that the success of farmed salmon in the natural environment is also determined by native population density [30,31,66]. It has been suggested that wild populations with lower densities (low population numbers) may be more at risk of the negative effects of hybridization and introgression from farmed fish [31,66,67]. Comparative studies within a natural setting are needed in order to further understand what drives the growth differences between wild and farmed salmon in the wild. Furthermore, comparative studies at more varied densities are encouraged in order to further elucidate the effects of density on growth differences between farmed and wild conspecifics.

Studies investigating the performance of hybrids are crucial for understanding how hybridization between farmed and wild conspecifics influences wild population dynamics. Farmed escapees can successfully interbreed with wild salmon, producing F_1_ hybrid offspring, and the subsequent performance of these hybrids will likely determine the future success of the wild population [56]. Here, the hybrid growth was observably more similar to their farmed parents than their wild parents, which may influence their subsequent fitness in the wild. The hybrids in this study were maternal half-siblings to the farmed fish; therefore, it is possible that maternal effects influenced growth patterns. It is important therefore to understand how hybrids respond to changing environmental conditions for future salmonid conservation and management, and to include reciprocal hybrids in order to differentiate between the effect of maternal egg size and the effects of domestication. Further studies, which investigate the performance of backcrosses and reciprocal hybrids with wild fish, will further elucidate the impacts of introgression on local population fitness.

## Supplementary Material

SUPPLEMENTARY TABLES AND FIGURES

## Data accessibility

The dataset supporting the conclusions of this article is available in the Dryad Digital Repository at http://dx.doi.org/10.5061/dryad.4c6b2.
