# Supplementary material for: Does density influence relative growth performance of farm, wild and F1 hybrid Atlantic salmon in semi-natural and hatchery common garden conditions?
Source: R Soc Open Sci. 2016 Jul 6;3(7):160152. doi: 10.1098/rsos.160152 (PMC4968464; doi:10.1098/rsos.160152)

**SUPPLEMENTARY TABLES AND FIGURES**

**Table S1: Family crosses for the experiment.**

| **Family** | **Dam** | **Sire** | **Group** |
| --- | --- | --- | --- |
| **1** | M1 | M9 | Farm |
| **2** | M1 | E11 | Hybrid |
| **3** | M2 | M10 | Farm |
| **4** | M2 | E12 | Hybrid |
| **5** | M3 | M11 | Farm |
| **6** | M3 | E13 | Hybrid |
| **7** | M4 | M12 | Farm |
| **8** | M4 | E14 | Hybrid |
| **13** | M7 | M15 | Farm |
| **14** | M7 | E17 | Hybrid |
| **17** | E1 | E11 | Wild |
| **18** | E2 | E12 | Wild |
| **19** | E3 | E13 | Wild |
| **20** | E4 | E14 | Wild |
| **23** | E7 | E17 | Wild |

**Table S2: Details of the microsatellite multiplexes used to assign the un-assignable individuals back to family.**

| **Multiplex** | **Primers** | **Dye** | **Allele Size** | **No. Alleles** | **Direction** | **Sequences** | **References** |
| --- | --- | --- | --- | --- | --- | --- | --- |
|  |  |  |  |  |  |  |  |
| **1** | SSsp2210 | 6FAM | 124-176 | 14 | F | AAG TAT TCA TGC ACA CAC ATT CAC TGC | Paterson et al. 2004 |
|  |  |  |  |  | R | CAA GAC CCT TTT TCC AAT GGG ATT C |  |
|  | SSspG7 | PET | 119-207 | 22 | F | CTT GGT CCC GTT CTT ACG ACA ACC | Patterson et al. 2004 |
|  |  |  |  |  | R | TGC ACG CTG CTT GGT CCT TG |  |
|  | SsaD144 | NED | 102-254 | 37 | F | TTG TGA AGG GGC TGA CTA AC | King et.al 2005 |
|  |  |  |  |  | R | TCA ATT GTT GGG TGC ACA TAG |  |
|  | Ssa202 | 6FAM | 230-298 | 18 | F | CTT GGA ATA TCT AGA ATA TGG C | O'Reilly et al. 1996 |
|  |  |  |  |  | R | GTT CAT GTG TTA ATG TTG CGT G |  |
|  | Sp2201 | PET | 227-367 | 33 | F | TTA GAT GGT GGG ATA CTG GGA GGC | Patersson et al. 2004 |
|  |  |  |  |  | R | CGG GAG CCC CAT AAC CCT ACT AAT AAC |  |
|  | SsaD157 | NED | 271-411 | 35 | F | ATC GAA ATG GAA CTT TTG AAT G | King et.al 2005 |
|  |  |  |  |  | R | GCT TAG GGC TGA GAG AGG AAT AC |  |
| **2** | Ssa289 | PET | 112-134 | 10 | F | CTT TAC AAA TAG ACA GAC T | McConnell et al. 1995 |
|  |  |  |  |  | R | GTC ATA CAG TCA CTA TCA TC |  |
|  | Ssa14 | NED | 134-146 | 6 | F | CCT TTT GAC AGA TTT AGG ATT TC | McConnell et al. 1995 |
|  |  |  |  |  | R | CAA ACC AAA CAT ACC TAA AGC C |  |
|  | Ssa171 | NED | 197-255 | 26 | F | TTA TTA TCC AAA GGG GTC AAA A | O'Reilly et al. 1996 |
|  |  |  |  |  | R | GAG GTC GCT GGG GTT TAC TAT |  |
|  | Sp2216 | 6FAM | 190-270 | 21 | F | GGC CCA GAC AGA TAA ACA AAC ACG C | Paterson et al. 2004 |
|  |  |  |  |  | R | GCC AAC AGC AGC ATC TAC ACC CAG |  |
|  | Sp1605 | PET | 216-268 | 22 | F | CGT AAT GGA AGT CAG TGG ACT GG | Paterson et al. 2004 |
|  |  |  |  |  | R | CTG ATT TAG CTT TTT AGT GCC CAA TGC |  |

**Table S3: Pair-wise comparisons of log weight conducted between groups and between treatments, and of the average egg size among groups.** The p values have been adjusted for multiple comparisons using a Tukey adjustment. The Significance column denotes the p values as significance codes whereby ‘***’ <0.0001, ‘**’ <0.001, ‘*’ <0.01, ‘.’ ≤0.5 and ‘ns’ denotes not significantly different.

| **Contrast** | **Estimate** | **Std. Error** | **z value** | **P value** | **Significance** |
| --- | --- | --- | --- | --- | --- |
| Hybrid - Farm | -0.09138 | 0.02071 | -4.413 | 3.62e-05 | *** |
| Wild - Farm | -0.33787 | 0.02216 | -15.245 | 1.00e-05 | *** |
| Wild - Hybrid | -0.24649 | 0.02254 | -10.936 | 1.00e-05 | *** |
| Hatchery Control - Hatchery Low | -0.03282 | 0.05549 | -0.591 | 0.9764 | ns |
| Hatchery High - Hatchery Low | -0.15115 | 0.05672 | -2.665 | 0.0593 | ns |
| Semi-natural Low - Hatchery Low | -0.40929 | 0.0585 | -6.997 | <0.001 | *** |
| Semi-natural High - Hatchery Low | -0.4667 | 0.05722 | -8.156 | <0.001 | *** |
| Hatchery High - Hatchery Control | -0.11833 | 0.05605 | -2.111 | 0.215 | ns |
| Semi-natural Low - Hatchery Control | -0.37647 | 0.05844 | -6.442 | <0.001 | *** |
| Semi-natural High - Hatchery Control | -0.43388 | 0.05613 | -7.729 | <0.001 | *** |
| Semi-natural Low - Hatchery High | -0.25814 | 0.05718 | -4.515 | <0.001 | *** |
| Semi-natural High - Hatchery High | -0.31554 | 0.05655 | -5.58 | <0.001 | *** |
| Semi-natural High - Semi-natural Low | -0.0574 | 0.0577 | -0.995 | 0.8577 | ns |
| Hybrid egg size – Farm egg size | -0.00063 | 0.00021 | -2.96 | 0.0087 | ** |
| Wild egg size – Farm egg size | 0.0056 | 0.00021 | 26.28 | <1e-04 | *** |
| Wild egg size – Hybrid egg size | 0.0063 | 0.00021 | 29.61 | <1e-04 | *** |

**Table S4: Full models investigating relationship between weight and egg size variation at the different treatments**. The variables in bold were retained in the final models for each treatment. Egg size is only retained in the semi-natural treatments.

|  |  |  | Random effects | | | | Fixed effects | | | | | |
| --- | --- | --- | --- | --- | --- | --- | --- | --- | --- | --- | --- | --- |
| **Model** | N | Response | Variable | Chi.sq | Chi  Df | P | Variable | Sum Sq | Num  Df | Den  Df | F | P |
| Hatchery Low | 416 | Log  Weight | **Family** | **243.58** | **1** | **<0.00** | Egg size | 0.016 | 1 | 12.97 | 1.24 | 0.29 |
|  |  |  |  |  |  |  | **Group** | **1.10** | **2** | **11.94** | **43.08** | **0** |
| Hatchery Control | 840 | Log  Weight | **Family** | **434.43** | **1** | **<0.00** | Egg size | 0.013 | 1 | 11.16 | 1.80 | 0.21 |
|  |  |  |  |  |  |  | **Group** | **1.57** | **2** | **12.04** | **38.76** | **0** |
| Hatchery High | 844 | Log  Weight | **Family** | **559** | **1** | **<0.00** | **Egg size** | **0.23** | **1** | **11.40** | **12.35** | **0.005** |
|  |  |  |  |  |  |  | **Group** | **3.36** | **2** | **11.21** | **88.81** | **0** |
| Semi-natural Low | 181 | Log  Weight | Family | 1.1 | 1 | 0.29 | **Egg size** | **0.86** | **1** | **NA** | **21** | **<0.00** |
|  |  |  |  |  |  |  | **Group** | **3.47** | **2** | **NA** | **42.39** | **<0.00** |
| Semi-natural High |  | Log  Weight | **Family** | **783.94** | **1** | **<0.00** | **Egg size** | **0.61** | **1** | **11.2** | **22.13** | **0.00006** |
|  |  |  |  |  |  |  | **Group** | **2.35** | **2** | **11** | **42.74** | **0** |

**Table S5: Full models investigating relationship between survival and egg size variation at the different treatments**. Egg size is only retained in the hatchery high density treatment.

| **Model** | **Variable** | **Estimate** | **SE** | **Z** | ***P value*** |
| --- | --- | --- | --- | --- | --- |
| Hatchery Low | Intercept | 2.5 | 0.18 | 14.04 | <2e-06 |
|  | Egg size | -0.006 | 0.18 | -0.035 | 0.97 |
| Hatchery Control | Intercept | 2.8 | 0.22 | 12.6 | <2e-06 |
|  | Egg size | 0.19 | 0.21 | 0.89 | 0.37 |
| Hatchery High | Intercept | 2.94 | 0.2 | 14.25 | <2e-06 |
|  | Egg size | 0.64 | 0.22 | 2.95 | **0.003** |
| Semi-natural Low | Intercept | 0.58 | 0.17 | 3.48 | 0.0005 |
|  | Egg size | 0.17 | 0.17 | 1.01 | 0.29 |
| Semi-natural High | Intercept | -1.48 | 0.17 | -8.75 | <2e-06 |
|  | Egg size | -0.19 | 0.17 | -1.14 | 0.254 |

**Figure S1: Average monthly water temperature for the indoor hatchery tanks and outdoor semi-natural tanks during the experimental period.** Water temperature was recorded daily and is presented as monthly mean + range.


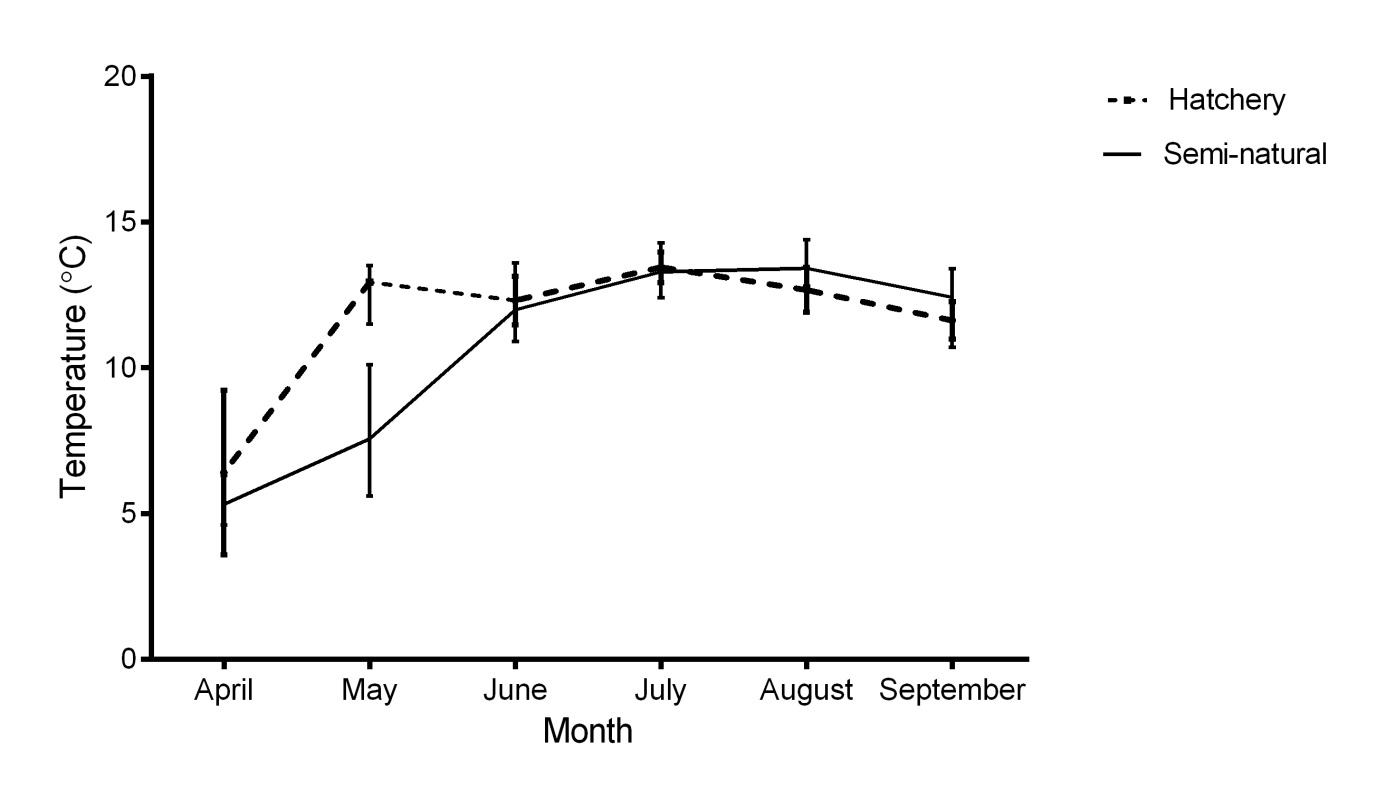

Supplement: SUPPLEMENTARY TABLES AND FIGURES [file rsos160152supp1.docx]
